# Application of Operational Load Monitoring System for Fatigue Estimation of Main Landing Gear Attachment Frame of an Aircraft

**DOI:** 10.3390/ma14216564

**Published:** 2021-11-01

**Authors:** Michal Dziendzikowski, Artur Kurnyta, Piotr Reymer, Marcin Kurdelski, Sylwester Klysz, Andrzej Leski, Krzysztof Dragan

**Affiliations:** 1 Airworthiness Division, Air Force Institute of Technology, ul. Ks. Boleslawa 6, 01-494 Warszawa, Poland; artur.kurnyta@itwl.pl (A.K.); piotr.reymer@wat.edu.pl (P.R.); marcin.kurdelski@itwl.pl (M.K.); sylwester.klysz@itwl.pl (S.K.); krzysztof.dragan@itwl.pl (K.D.); 2 Faculty of Mechanical Engineering, Military University of Technology, ul. gen. S. Kaliskiego 2, 00-908 Warszawa, Poland; andrzej.leski@wat.edu.pl; 3 Faculty of Technical Sciences, University of Warmia and Mazury in Olsztyn, ul. M. Oczapowskiego 2, 10-719 Olsztyn, Poland; 4 Institute of Aviation, Lukasiewicz Research Network, al. Krakowska 110/114, 02-256 Warszawa, Poland

**Keywords:** aircraft load monitoring, fatigue estimation, structural health monitoring, landing gear monitoring

## Abstract

In this paper, we present an approach to fatigue estimation of a Main Landing Gear (MLG) attachment frame due to vertical landing forces based on Operational Loads Monitoring (OLM) system records. In particular, the impact of different phases of landing and on ground operations and fatigue wear of the MLG frame is analyzed. The main functionality of the developed OLM system is the individual assessment of fatigue of the main landing gear node structure for Su-22UM3K aircraft due to standard and Touch-And-Go (T&G) landings. Furthermore, the system allows for assessment of stress cumulation in the main landing gear node structure during touchdown and allows for detection of hard landings. Determination of selected stages of flight, classification of different types of load cycles of the structure recorded by strain gauge sensors during standard full stop landings and taxiing are also implemented in the developed system. Based on those capabilities, it is possible to monitor and compare equivalents of landing fatigue wear between airplanes and landing fatigue wear across all flights of a given airplane, which can be incorporated into fleet management paradigms for the purpose of optimal maintenance of aircraft. In this article, a detailed description of the system and algorithms used for landing gear node fatigue assessment is provided, and the results obtained during the 3-year period of system operation for the fleet of six aircraft are delivered and discussed.

## 1. Introduction

Developments of the industrial revolution, in particular rapid growth of rail transport, led to the discovery that failure of the material can occur under a stress level much lower than static tensile strength [1]. Limited durability of structural elements was the main driver for the pioneer experiments carried out among others by Wöhler in the field of S–N curves or Palmgren and Miner who developed the linear damage cumulation—which provided the basis for estimating safe-life of structures [2]. In aerospace, the beginnings of system solutions focused on a high level of safety date back to the early post-war years, when the safe-life design concept was introduced, in order to preserve human health and life as well as mitigating potential material losses [2,3]. Due to the advancement of aircraft structures, complexity of different types of connections and variable geometry of structural elements, the most reliable method for determining the service life is the subcomponent or full scale fatigue tests of aircraft structures [4,5,6]. After the F-111 aircraft crash in 1969 [7,8], a new design and maintenance concept was introduced—the damage tolerance approach [9,10]. In the new methodology, a mandatory requirement for assuring structural integrity is the introduction of Non-Destructive Testing (NDT) procedures and definition of inspection intervals of aircraft critical structural elements [11]. Non-destructive inspections were proposed in order to assure possibility of damage detection, before their development could jeopardize the safety of aircraft operation. Introduction of the damage tolerance concept resulted in significant decrease of the risk of air accidents due to fatigue damage. Despite remarkable advances in the field of fracture mechanics and the development of numerical modeling methods for complex physical processes, it is impossible to predict all factors that may increase the risk of an accident. In particular, the design load spectrum of the structure determines the intervals between subsequent NDT inspections as well as safety margins for aircraft operation. However, the way in which a particular aircraft is operated after its introduction into service does not necessarily fit to its pre-assumed profile, especially for combat aircraft, therefore the load and fatigue spectra can change during long-term operational use due to numerous factors [12]. Thus, it is necessary to introduce an Individual Aircraft Tracking (IAT) program in order to monitor actual load spectrum of every aircraft in operation [10,13,14]. A modern approach to meet this requirement is to implement Operational Loads Monitoring (OLM) systems [15] as a necessary component of aircraft avionics. The approach for this purpose has evolved from g-counters to modern on-board parametric systems incorporating several aspects, e.g., material fatigue, measurement techniques, signal analysis, modeling, etc., and utilizing a network of sensors, e.g., strain gauges or Fiber Bragg Gratings (FBG) [16,17,18,19] which are permanently mounted in the aircraft structure and measure local strain in predefined aircraft locations. Regarding the above, introduction of an Individual Aircraft Tracking (IAT) program with use of proper OLM system is essential to ensure safety and extend aircraft service life [13,14,20,21]. Development of OLM systems can also significantly increase potential use of ageing aircrafts [12], as after performing the necessary overhaul, such technology may allow their further operation beyond initially designed service life, if fatigue wear or condition of critical structures can be precisely monitored based on indications from network of integrated sensors.

In this article, an approach to fatigue estimation of the Main Landing Gear (MLG) attachment frame due to vertical landing forces based on Operational Loads Monitoring (OLM) system developed for Su-22UM3K aircraft is presented. In Section 2, the approach to OLM system design is presented and algorithms for signal analysis and fatigue assessment are defined. In Section 3 the results obtained during the 3-year period of system operation for the fleet of six aircraft are delivered and discussed, and in the last section the article is concluded.

## 2. Methodology for Load Analysis of Landing Gear Attachment Node

### 2.1. Full Scale Fatigue Test and Load Monitoring System of Su-22 Aircraft

Su-22 is a variable wing sweep angle fighter–bomber aircraft which was introduced into operation in Polish Armed Forces (PLAF) in mid-1980’s. There are two versions of the aircraft: single-seated combat version M4 and a two-seated trainer version UM3K. Based on the PLAF decision, a service life extension program was launched in 2014 in order to prolong the designed operation period guaranteed by the manufacturer. As some of the aircraft were close to the original limits, the actual service life, adequate to the flight profile in Polish Air Force, had to be validated. Full-Scale Fatigue Tests (FSFTs) have been carried out in order to verify that the required service life is available with respect to adequate load spectrum [22]. Furthermore, for two-seated Su22UM3K aircraft, the development of an OLM system providing reliable and detailed data for Individual Aircraft Tracking (IAT) program was required, due to their increased usage compared to M4 version, in particular an increased number of standard and Touch-And-Go landings [14].

**Figure 1:**
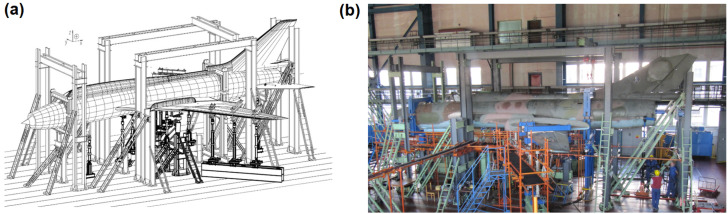
Full-Scale Fatigue Test rig: (**a**) schematic view and (**b**) actual view.

FSFT was designed to be a four-stage test due to the variable sweep angle of the aircraft wings [22]. This was decided in order to minimize the laborious and time consuming wing sweep changes and actuator reconfigurations. Different wing sweep angles are used for takeoff and landing (30${}^{{}^{\circ}}$), subsonic flights (45${}^{{}^{\circ}}$) and supersonic flights (63${}^{{}^{\circ}}$). The final goal of the FSFT was determination of the total durability of the structure in order to reach 3200 flight hours and 6000 landings. Based on analysis of flight profiles of Polish Su-22, four aircraft configurations for subsequent stages were determined (Table 1). Landing loads and loads for flight with extended flaps were represented in separate stages due to different hydraulic actuators arrangement under wings. The final stage was designed to represent flights with the highest vertical overloads from Stage 2 until appearance of critical damage of the structure. In order to represent the load distribution in each stage, an array of hydraulic actuators was designed for each stage (Figure 1) taking into account load and displacement range for each considered load node as well as wing sweep angle and aircraft configuration.

**Figure 2:**
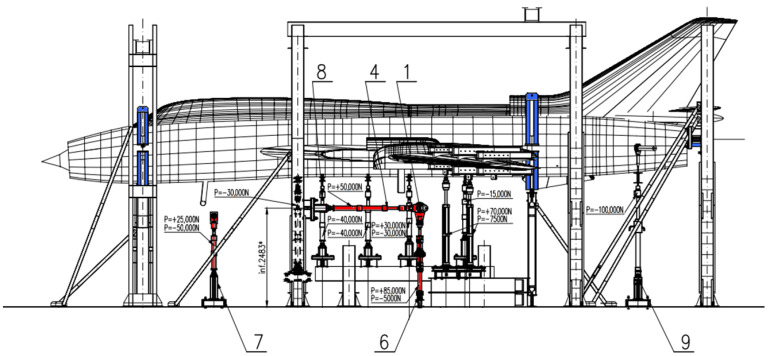
Configuration of actuators for Stage I of Full-Scale Fatigue Test execution.

**Figure 3:**
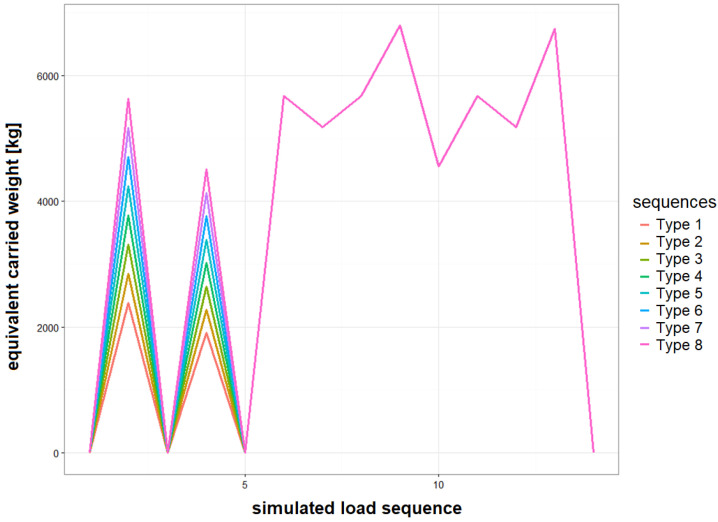
Simulated load sequences exerted on landing gear attachment node during the Full-Scale Fatigue Test representing different types of full stop landings.

This article is devoted to the development of a methodology for fatigue estimation of Main Landing Gear (MLG) attachment frame due to aircraft landing; therefore, we will focus on Stage I of the test while more detailed description of the approach to FSFT of Su-22 aircraft and other test Stages can be found in [22,23]. For Stage I, the wing sweep was set to 30° and the structure was fixed with clamps on the fourth and thirty-fourth fuselage frame as well as by two rods mounted in the engine bay (highlighted in blue in Figure 2). Loads were exerted on the structure by means of 16 hydraulic actuators. Six actuators (highlighted in red in Figure 2) were used in order to represent front (1 actuator acting in the vertical direction) and main landing gear loads (2 actuator on each side representing vertical and longitudinal landing forces and 1 actuator in the middle representing transverse taxing forces), whereas 4 actuators were acting on the variable sweep wing part (2 each side) corresponding to lift and inertia forces during landing. The remaining 6 actuators were distributed along the fuselage in order to represent inertial forces. In this study, fatigue wear of the attachment frame due to it being carried by MLG is considered as one of the key factors determining remaining service life of an aircraft. Those forces were represented by actuators denoted as no. 6 in Figure 2 (one actuator on each side). Different types of full stop landings were represented during Stage I with the same landing mass of the aircraft but different vertical load levels during touchdown. Simulated vertical load sequences on the actuators in terms of the equivalent weight carried by main landing gear node is shown in Figure 3, and the number of different type of landings represented in FSFT load spectrum is provided in Table 2 below. Each full stop landing was represented by 14 load levels exerted on the frame of the aircraft in linear sequences (Figure 3).

**Figure 4:**
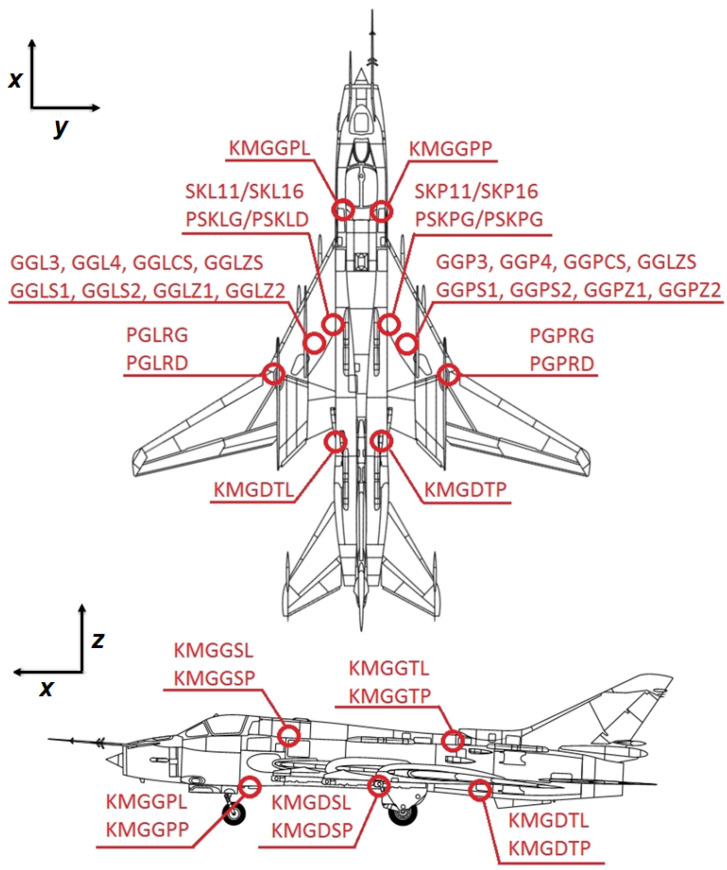
General view of the strain gauge network on the test aircraft (due to the symmetry of the sensor network only one side of the aircraft is shown).

Load envelope and landing statistics were estimated based on dedicated flight tests program and historical flight data. The information about stress level occurring in various areas of interest during flight was collected with use of a network of strain gauges installed on a test aircraft. After sensor installation, a dedicated flight test program was performed in order to acquire sufficient data about load distribution during flight for the purpose of FSFT load spectra preparation for different test stages. In total, 40 strain gauges were installed on the test aircraft selected for in flight measurements (Figure 4):

- 15 on the left wing (and symmetrically on the right wing):
  - 4 on the wing fuselage joint,
  - 2 on the main pivot joint,
  - 9 on main landing gear and in the compartment and
- 10 on the fuselage in 5 selected Sections (2 each).

The same installation was used for test structure monitoring during FSFT.

The strain gauge measurement array was designed to monitor the following loads:

- bending momentum in the wing fuselage joint (one section each side);
- bending momentum in the main pivot joint (one section each side);
- bending momentum in the fuselage (5 sections);
- main landing gear loads:
  - vertical force along *z* axis;
  - bending momentum along *y* axis;
  - bending momentum along *x* axis.

**Figure 5:**
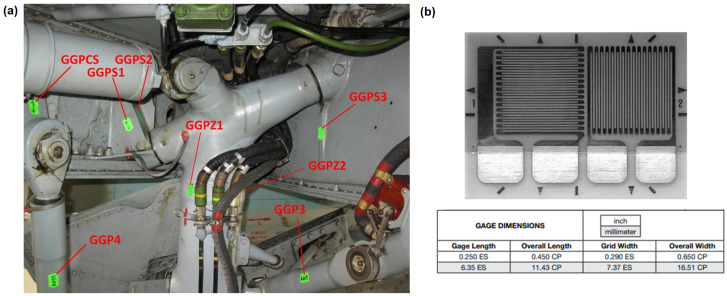
Localization and type of strain gauges installed on test aircraft: (**a**) localization of sensors in MLG compartment and (**b**) tee rosette strain gauges used (250UT manufactured by Vishay).

All of the strain gauges were in the form of tee rosette configuration (Figure 5b), which allowed for strain measurements in the primary direction with temperature compensation due to the secondary perpendicular strain gauge. In Figure 5a, localization of strain gauges installed in the main landing gear compartment is presented.

In addition, a load monitoring system with a reduced number of strain gauges was installed on six selected Su-22UM3K aircraft, in order to track their individual usage and remaining service life. In particular, as aircraft selected to be equipped with a Operational Load Monitoring (OLM) system are used for pilot training and certification, it was necessary to develop a methodology for main landing gear attachment node fatigue wear monitoring, as those aircraft perform more landings (especially Touch-And-Go landings) than combat one-seat aircraft. There exist many approaches to Individual Aircraft Tracking [10] depending on the type of data available for load estimation in critical locations. A through revision of different methods for fatigue assessment and comparison of different monitoring techniques is provided in [24]. One of the most common approaches is to use the vertical overload parameter recorded by Flight Data Recorder (FDR) in order to determine stress levels at critical locations. This parameter, in particular in combination with some other flight parameters, e.g., angle of attack and aircraft mass distribution, is especially efficient for fatigue assessment of wing spars or wing to fuselage attachment nodes, as vertical acceleration is the key parameter determining fatigue wear of such structural elements. Based on acceleration measurements, it is moreover possible to define many useful damage sensitive signal features for the purpose of Structural Health Monitoring [25]. However, the bandwidth and sensitivity of accelerometers used in FDR are sometimes not sufficient for proper determination of fatigue due to landings, in particular it can be hard to detect and properly assess touchdown of an aircraft based on that parameter. Furthermore, accelerometers cannot be used for determination of takeoff weight which in the case of landing gear attachment node can significantly contribute to Ground-Air-Ground cycle, therefore strain gauges were used for OLM purposes.

**Figure 6:**
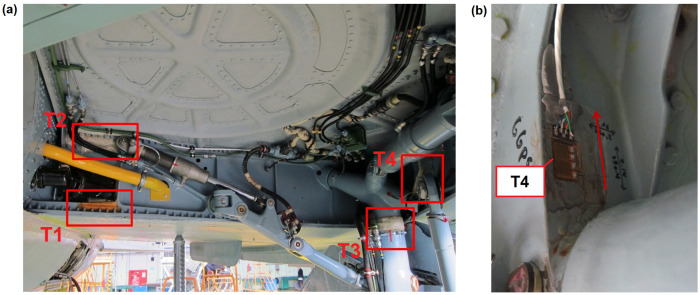
Reduced system for load monitoring: (**a**) general location of strain gauges of the system (left MLG bay) and (**b**) view of T4 sensor with indication of measurement direction.

The sensor network of the system developed for the IAT program is reduced with respect to the system installed on the test aircraft (Figure 4). It contains eight strain gauges which are installed symmetrically in the most relevant structural elements of the aircraft on both wings and main landing gear frame. In Figure 6 general location of strain gauges of the reduced system is presented. The limitation to eight strain gauges was due to the hardware requirements of the FDR used. Four sensors are placed on the main wing spar, on lower (sensors denoted as T1 and T5) and upper (sensors denoted as T2 and T6) flanges. For main landing gear monitoring, the sensor denoted as GGPZ1 in Figure 5a was selected to measure backward bending moment of the landing gear (denoted as T3 and T7 in the reduced system), and sensor GGPS2 (Figure 5a) is used for estimation of vertical force during landing (denoted as T4 and T8 in the reduced system). Strain gauge selection was determined based on signal-to-noise ratio measured during flight tests as well as the results of sensors calibration on test specimen during FSFT. All the selected strain gauges depended linearly on the corresponding relevant forces. In particular, sensor T4 (Figure 6b) was linearly dependent on vertical force applied to main landing gear in full range of the load spectrum while it was barely sensitive to force applied in perpendicular direction. Therefore, for the purpose of fatigue wear estimation of the MLG attachment node due to vertical landing forces, the strain values ε measured by sensors T4 (left node) and T8 (right node) were used.

### 2.2. Methodology of Signal Analysis for Landing Operations Extraction

**Figure 7:**
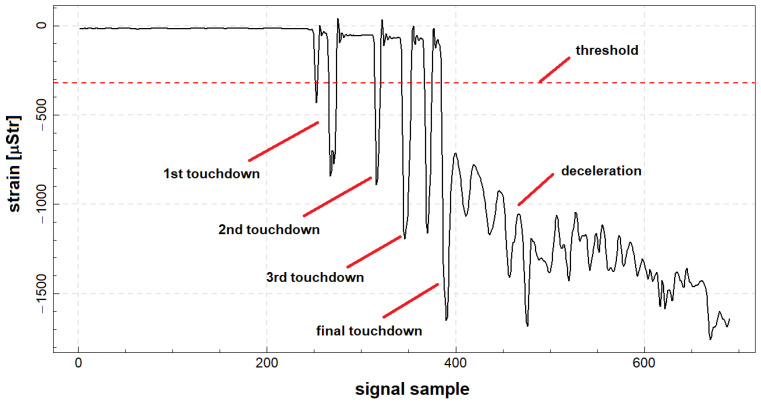
An example of signal acquired by strain gauge located at main gear attachment node during full stop landing with indication of signal threshold for landing detection.

In order to determine fatigue of landing gear attachment node, it is necessary to extract signals corresponding to on-ground operations from full record of a given flight. For the detection of on-ground segments recorded by the system, it was assumed that the threshold $\varepsilon_{T}$ for detection of significant deformations originating from landing gear loads corresponding to landings is 20% of the base deformation, which is determined before the takeoff of the plane, using the loads at full stop. In Figure 7, an example of signal acquired during landing with multiple touchdowns preceding final touchdown and deceleration is presented. As can be seen, signal can exceed the estimated threshold during before final deceleration and load transfer to landing gear node; therefore, for a proper determination of signal corresponding to landings, an additional algorithm is required.

In the presented algorithm, the first step of on-ground operations detection and classification is to select all the signal samples ε(t) below the determined threshold $\varepsilon_{T}$, i.e., satisfying the condition

(1) $$\varepsilon \left(t\right)<\varepsilon_{T},$$

where *t* is time of a given sample acquisition. Then, starting from the first signal sample satisfying this condition, consecutive disjoints sets of data are determined:

(2) $$\tau_{1}=\left\{\varepsilon \left(t_{i,1}\right),\ldots \varepsilon \left(t_{f,1}\right)\right\},\ldots ,\tau_{l}=\left\{\varepsilon \left(t_{i,l}\right),\ldots \varepsilon \left(t_{f,l}\right)\right\},$$

such that the difference between the acquisition time $t_{i,k}$ of the initial signal value $\varepsilon (t_{i,k})$ from a given set $\tau_{k}$ and acquisition time $t_{f,k-1}$ of the final signal value $\varepsilon (t_{f,k-1})$ from the preceding set $\tau_{k-1}$ is not less than 3 s. For proper on-ground connected signal segments detection, datasets $\tau_{j}$ lasting no longer than 2 s, i.e., for which

(3) $$t_{f,j}-t_{i,j}\leq 2$$

are disregarded, in order to remove eventual artificial events due to natural signal disturbances, e.g., due to short power outages or drops of pressure in hydraulic blocks during flight. In the next step, for every segment $\tau_{j}$, all the signal samples ε(t) satisfying

(4) $$t_{i,j}-3\leq t\leq t_{f,j}+3$$

are added to a given set $\tau_{j}$ in order to analyze full data records corresponding to subsequent on-ground operations, i.e., each part of the signal corresponding to such operation is extended by additional 3 s time offset of signal before and after the operation, in order to track all load cycles exerted on landing gear node. The first of such extended sets, $\tau_{1}$, contains signal acquired during aircraft takeoff and taxiing before takeoff, and the last set $\tau_{l}$ corresponds to full stop landing and taxiing after landing, whereas all sets in between are classified as Touch-And-Go (T&G) landings.

**Figure 8:**
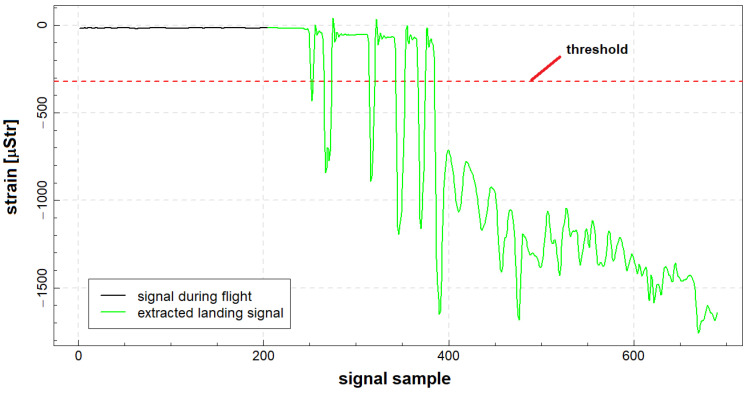
An example of automated extraction of signal corresponding to full stop landing.

In Figure 8, the outcome of the proposed algorithm for landing presented in Figure 7 is delivered. The signal corresponding to full stop landing was extracted from raw signal with use of the proposed algorithm. In particular, all the aircraft touchdowns are included in the extracted signal, and an additional 3 s time offset provides data corresponding to no load condition on landing gear node; therefore, based on such data, it is possible to capture all relevant load cycles exerted on the node due to landing.

All the parameters, e.g., threshold $\varepsilon_{T}$ and offset levels, were decided based on algorithm results on database of reference signals, where signals corresponding to on-ground operations were manually determined, so the number and duration of landings was compared between expert and automated analysis for different adjustments of the parameters. Furthermore, the performance of the algorithm is periodically verified, i.e., number of landings is compared with maintenance data, but also validity and performance of the algorithm is evaluated by the experts based on randomly selected flights from a given period.

**Figure 9:**
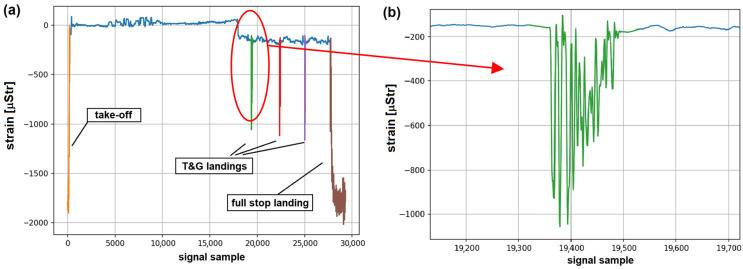
An example of load monitoring system records: (**a**) full record with indication of all detected on-ground operations; (**b**) signal acquired during first detected Touch-And-Go landing (marked in green).

**Figure 10:**
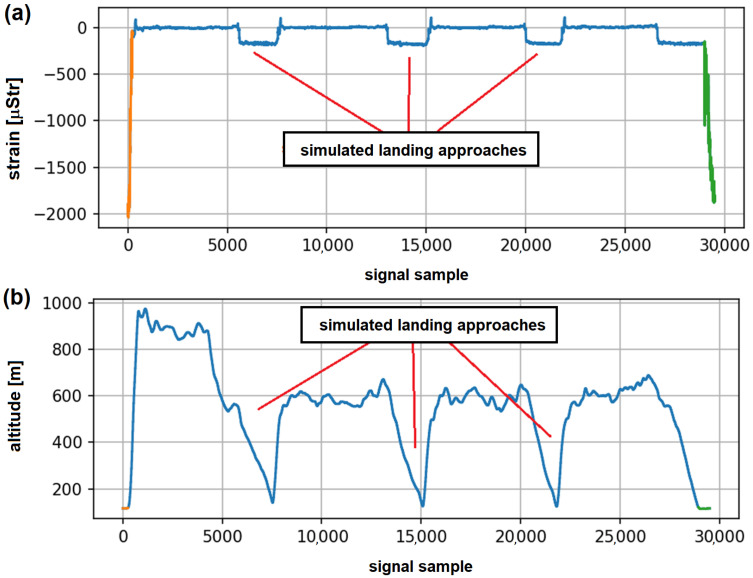
An example of flight records with simulated landing approaches: (**a**) strain on landing gear attachment node with indication of take-off and full stop landing; (**b**) altitude (barometric).

In Figure 9a, an example of an OLM system record with indication of subsequent on-ground operations is presented. The approach is efficient in detection of on-ground operations, in particular for Touch-And-Go landings (Figure 9b). Detection of such events based on flight parameters acquired by a standard flight data recorder was very inaccurate, as many simulated landing approaches, but without touchdown, are performed during pilot training process. In Figure 10, an example of system records for a flight with three simulated attempts to landing is presented. For every such maneuver, low pass flight over runway was performed (Figure 10b) with released landing gear, which caused a slight change of strain values recorded on the node (Figure 10a); however, no actual touchdown occurred. This is correctly recognized by the proposed algorithm, as only takeoff and full stop landing were detected in that flight (Figure 10a). As both records—the altitude parameter as well as indicator of landing gear release command—were similar as for proper Touch-And-Go landings, such events were often misclassified with use of algorithms based on only flight parameters records, as sensitivity and signal sampling rate of the standard g-load sensor used on this type of aircraft are not sufficient for such events detection.

**Figure 11:**
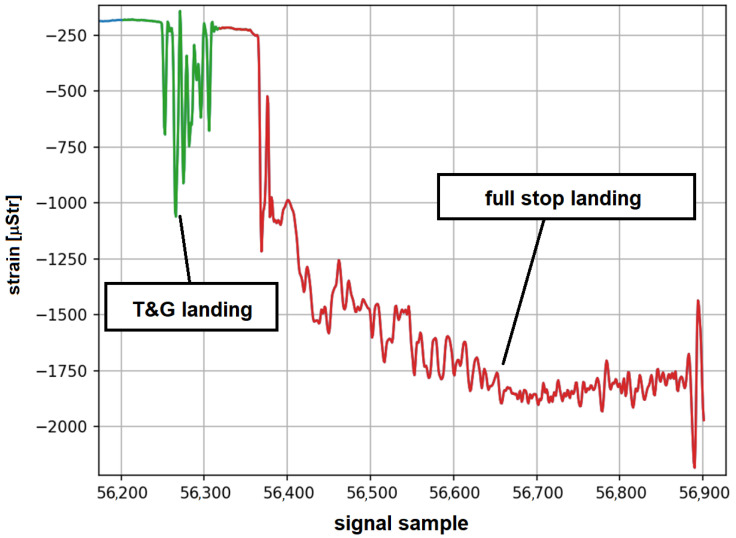
An example of system record with subsequent Touch-And-Go and full stop landing.

An interesting example of algorithm output is presented in Figure 11. The recorded strain signal shows a clear example of a bounced landing. In this case, after the first touchdown, the plane took off from the ground for about two seconds, and full stop landing was performed afterwards. The algorithm detected a Touch-And-Go landing event and subsequent proper landing. Such events, when detected by the system with high confidence, can provide an automated tool for human error assessment, which could be beneficial for pilot training programs.

**Figure 12:**
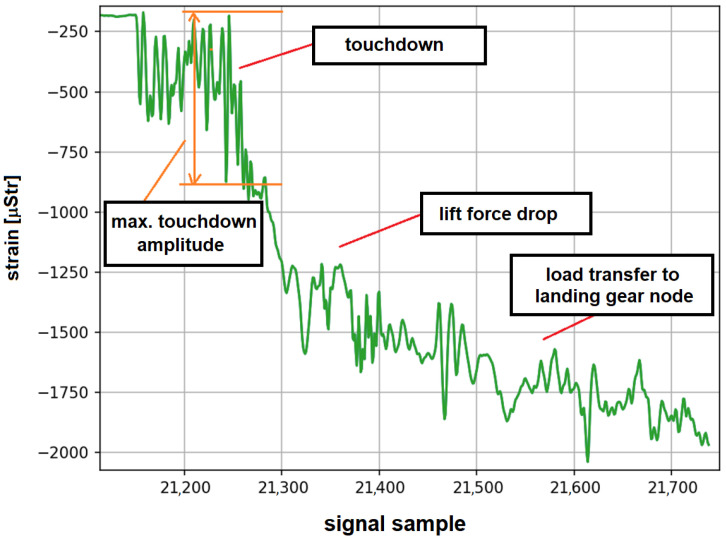
An example of strain record for full stop landing with indication of subsequent stages of this maneuver.

In Figure 12, an example of a strain gauge signal recorded during full stop landing is shown, with indication of the characteristic stages of this process. One of the parameters used to assess the stress level of the main landing gear node structure during full stop and Touch-And-Go landings is the amplitude of the maximum deformation cycle recorded during the touchdown (Figure 12). A characteristic feature of the touchdown is the rapid deformation change related to the slowing down of the descent speed of the aircraft and the dissipation of energy on the elements of the landing gear node [26]. Two warning levels were defined for the strain signals characterizing hard landings, which are presented in Table 3. In addition to structural load monitoring purposes, the distribution of landings corresponding to different warning levels can also be used for the assessment of pilot training advancements. The warning levels were determined based on landing statistics determined for a certain period of time. Another approach could be based on relevant material data and depend on the stress values at the critical point for the main landing gear attachment node, yet the adopted statistical approach is better suited for pilot training purposes.

### 2.3. Methodology of Landing Gear Node Fatigue Estimation

In this paragraph, a method for fatigue estimation of Main Landing Gear (MLG) attachment frame due to vertical landing forces based on OLM system records is delivered. The classic approach to fatigue estimation is based on the S–N fatigue curve of a given material and linear cumulative Palmgren–Miner hypothesis. The S–N curve determines the relationship between the equivalent stress amplitude $\sigma^{eq}$ of the zero-to-tension load cycle and the number of cycles *N* needed to fracture of an element, subjected to load cycles of this amplitude. The S–N curve is usually described by the relationship [27]

(5) $$\log\sigma^{eq}=A+B\log N.$$

Cumulative fatigue *D* of a set of load cycles $\left\{\sigma_{1}^{eq},\ldots ,\sigma_{M}^{eq}\right\}$ is given by the expression

(6) $$D=\sum_{i=1}^{M}\frac{1}{N_{i}}=C\sum_{i=1}^{M}{\left(\sigma_{i}^{eq}\right)}^{-\frac{1}{B}},$$

where *C* is a constant dependent on material constants *A* and *B*.

Based on classic S–N and cumulative fatigue equations presented above, an approach to fatigue wear estimation of main landing gear attachment frame can be defined as follows. In order to determine the fatigue wear of a given structural element, a linear relation between stress level $\sigma^{c}$ at critical point of the element and some physical parameter *p* can be assumed:

(7) $$\sigma_{c}=\alpha p.$$

As the parameter *p*, records of strain gauge ε installed in a point of the structure where stress level is linearly proportional to $\sigma^{c}$ can be used, or *p* can be a function of flight parameters determining load values of a given structural element, e.g., weight and *g*-load factor in the case of fuselage wing attachment. Equation (6) can be rewritten as follows:

(8) $$D=C\sum_{i=1}^{M}{\left(\sigma_{c,i}^{eq}\right)}^{-\frac{1}{B}}=\tilde{C}\sum_{i=1}^{M}p_{i}^{-\frac{1}{B}},$$

where $\tilde{C}=C\alpha^{-\frac{1}{B}}$ and $p_{i}$ is value of physical parameter corresponding to equivalent zero-to-tension stress amplitude of the *i*-th load cycle.

The material constant *C* and proportionality parameter α can be omitted if reference fatigue for a given set of cycles is known, for instance, if data from Full Scale Fatigue Test are available. In that case,

(9) $$1=D_{FSFT}=\tilde{C}\sum_{j=1}^{M_{FSFT}}p_{j}^{-\frac{1}{B}},$$

where summation is carried over load cycles $p_{j}$ exerted on the structure during Full-Scale Fatigue Test (FSFT) and $M_{FSFT}$ is number of load cycles during FSFT. Therefore,

(10) $$D=\frac{D}{D_{FSFT}}=\frac{\sum_{i=1}^{M}p_{i}^{-\frac{1}{B}}}{\sum_{j=1}^{M_{FSFT}}p_{j}^{-\frac{1}{B}}},$$

can be calculated if material constant *B* and reference data from FSFT is known.

There exist several conditions which need to be satisfied in order to apply the presented approach based on Miner’s law. First, if only laboratory material data are available with no reference fatigue exerted on a real test structure, then the correspondence between stress level in the critical location of the structure and the parameter used for fatigue evaluation must be known in exact form and Miner’s Equation (6) needs to be used directly instead of the Equation (10). Another requirement is a linear relation between the physical parameter used for fatigue assessment and stress level $\sigma_{c}$ in the critical location. As mentioned in Section 2.1, strain gauge readings used in the study are linearly dependent on vertical landing force in full range of admissible loads of the aircraft, as confirmed during FSFT rig calibration. Furthermore, in the case of Su-22 aircraft, it is assumed in load spectra design that the aircraft will be operated within the linear elastic regime of the materials, as if high overloads during flight or very harsh landings occur, special procedures are introduced in order to evaluate aircraft condition (e.g., plastic deformations) and its airworthiness. In addition, no looseness in highly loaded joints is allowed, therefore linearity between vertical force and stress level in critical location is legitimate assumption in our case. Finally, fatigue limit [1] of the material needs to be considered for fatigue estimation based on Equation (10), as load cycles below the fatigue threshold in a given point can be above fatigue limit in critical location. In our case, all strain data corresponding to physical load cycles exerted on main landing gear attachment are taken into account for the purpose of fatigue assessment, therefore all the load cycles in critical location are considered as well.

For the purpose of this study, the equivalent weight *w* carried by landing gear node was adopted as a physical parameter needed for fatigue wear calculation in accordance with the Equation (8). Equivalent weight was assessed by strain gauge reading and results of linear physical scaling, i.e., values of strain for lifted aircraft with released landing gear were related to no load condition and values of strain readings for aircraft on-ground were related to its measured weight carried by a given landing gear node. Furthermore, data from actuators used for the Full-Scale Fatigue Test were rescaled in those units and used in the denominator of the Equation (10) for fatigue calculation. The parameter *B* was estimated based on laboratory fatigue tests of material used for landing gear attachment node manufacturing.

For easier interpretation of the obtained results, a notion of Landing Fatigue Equivalent (*LFE*) can be introduced. *LFE* represents the relative fatigue of landing gear node due to loads exerted during a given landing $D_{l}$ with respect to mean fatigue due to simulated landings during FSFT:

(11) $$LFE=\frac{\sum_{i=1}^{M_{l}}w_{i}^{-\frac{1}{B}}}{\frac{1}{N}\sum_{j=1}^{M_{FSFT}}w_{j}^{-\frac{1}{B}}},$$

where:

- $w_{i}$ denotes amplitude of equivalent zero-to-tension load cycles exerted on landing gear node during landing (in terms of equivalent weight carried by the node as measured by strain gauge),
- $M_{l}$ denotes number of load cycles recorded during landing,
- $w_{j}$ denotes amplitude of equivalent zero-to-tension load cycles exerted on landing gear node during FSFT (in terms of equivalent weight carried by the node) and
- *N* denotes number of simulated landings during FSFT.

**Figure 13:**
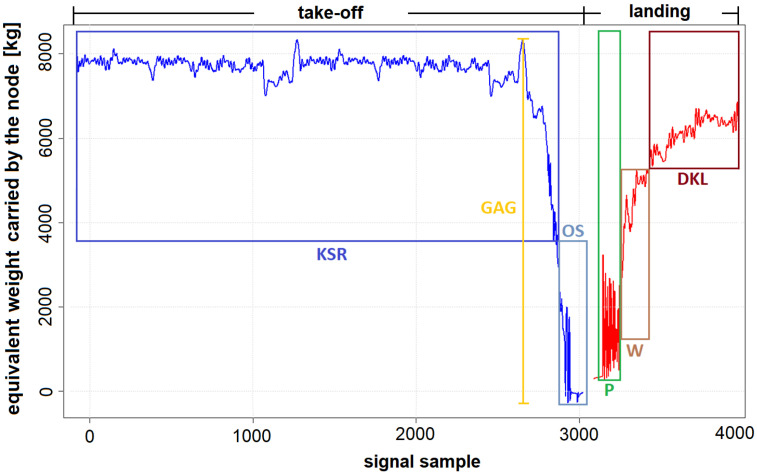
An example of strain record for full stop landing with indication of subsequent stages of this maneuver.

In order to account properly Ground–Air–Ground cycle when calculating *LFE* for full stop landing, the records of strain gauge obtained for takeoff and landing are joined (Figure 13) prior to determination of load cycles with use of Range-Pair Counting algorithm [28]. Furthermore, the noise level of the recorded signal during on-ground operations was estimated, and for *LFE* calculation, only relevant recorded cycles, i.e., corresponding to physical load of the structure and higher than the level of noise, were considered. *LFE* provides a direct measure to estimate the fatigue wear of the landing gear attachment node for a given flight if:

- LFE<1 then loads exerted on landing gear attachment node during a given landing was less severe than mean landing profile during FSFT; or
- LFE>1 then loads exerted on landing gear attachment node during a given landing was more severe than mean landing profile during FSFT.

Cumulative *LFE* obtained for a given aircraft can be considered as a limiting condition for possibility of further aircraft operation instead of total estimated fatigue for landing gear attachment.

For Touch-And-Go landings, all the load cycles are due to aircraft touchdown. For full stop landing, additional information about this process can be obtained by distinction of load cycles occurring during different stages of landing (Figure 12) and calculation of the corresponding fatigue. An algorithm for the classification of structure load cycles recorded by strain gauges during the normal landings and other ground loads during take-off and full stop landing was developed. It was assumed that the cycles are classified into the following categories:

- cycles recorded during touchdown—P;
- Ground–Air–Ground cycle—GAG;
- cycles recorded during braking—W;
- cycles recorded during taxing before take-off—KSR;
- cycles recorded during taxing after landing—DKL;
- other cycles recorded during takeoff—OS;
- other cycles (not classified elsewhere)—O.

In Figure 13, the general concept of division of a single take-off and landing operation into above defined cycles is shown—the height of the shown color boxes correspond the assumed minimum and maximum values of certain types of cycles for a given flight.

Since:

(12) $$\begin{array}{cc} \sum_{i=1}^{M_{l}}w_{i}^{-\frac{1}{B}} & =w_{GAG}^{-\frac{1}{B}}+\sum_{c\in P}w_{c}^{-\frac{1}{B}}+\sum_{c\in W}w_{c}^{-\frac{1}{B}}+\sum_{c\in KSR}w_{c}^{-\frac{1}{B}}+\sum_{c\in DKL}w_{c}^{-\frac{1}{B}}+\\ \; & +\sum_{c\in OS}w_{c}^{-\frac{1}{B}}+\sum_{c\in O}w_{c}^{-\frac{1}{B}}, \end{array}$$

where *c* denotes cycles corresponding to different types, also fatigue equivalent obtained for full stop landing can be factored accordingly:

(13) $$\begin{array}{cc} LFE & =LFE_{GAG}+LFE_{P}+LFE_{W}+LFE_{KSR}+LFE_{DKL}+LFE_{DKL}+\\ \; & +LFE_{OS}+LFE_{O}. \end{array}$$

Based on investigation of the defined contributions to fatigue equivalent, additional conclusions can be drawn, e.g., with respect to pilot training program or with respect to condition of the runway, as will be shown further in the text.

## 3. Results

**Figure 14:**
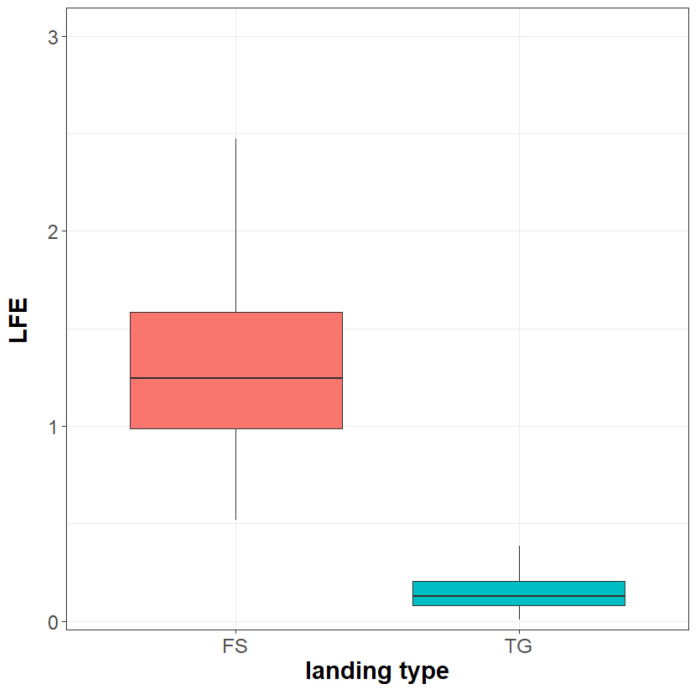
Boxplot of Landing Fatigue Equivalent for full stop (FS) and Touch-And-Go (T&G) landings.

In the period 2018–2021, a total number of 1563 full stop landings and 350 Touch-And-Go landings of six Su-22 aircraft equipped with Operational Load Monitoring system were recorded. In Figure 14, boxplots of *LFE* for full stop landing and Touch-And-Go landings are presented.

It is demonstrated that the fatigue wear of the landing gear attachment node is significantly higher for full stop landings compared to Touch-And-Go landings. In the former case, the median value of *LFE* is 1.24 compared to 0.13 obtained for the latter landings type. This result is of particular importance for estimation of remaining fatigue life of landing gear attachment node for aircraft not equipped with a load monitoring system, as usually service life is evaluated with respect to number of landings without distinction between full stop and Touch-And-Go landings. Median value of fatigue corresponding to full stop landing is about 24% higher mean fatigue value of simulated landings represented during Full-Scale Fatigue Test. Partially, it can be related to number of touchdown cycles represented for simulated landings. For simulated landings, only two load cycles were represented for touchdown as shown in Figure 3, in order to meet time constraint requirements for the test completion. In reality, the number of load cycles during aircraft touchdown is significantly higher as can be seen in Figure 9b or Figure 12.

In Table 4, the distribution of *LFE* to a different type of fatigue cycle is presented. Higher fatigue wear of the landing gear node obtained for full stop landing is mostly due to the Ground–Air–Ground cycle. The median value of the *LFE* for the GAG cycle is 1.04, which is approximately 84% of total median fatigue equivalent. This indicates that increased simulated take-off weight of aircraft during FSFT (Figure 3) would represent better true distribution of operational take-off aircraft weight. Considerable contribution to *LFE* is also due to touchdown loads, i.e., median $LFE_{P}=0.083$.

**Figure 15:**
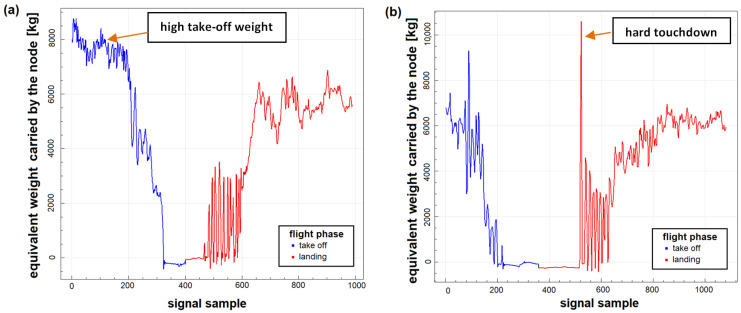
An example of system records for flights with high values of Landing Fatigue Equivalent obtained for full stop landing: (**a**) flight with increased take-off aircraft weight; (**b**) flight with hard touchdown.

Typically, higher *LFE* is due to increased takeoff weight or due to hard touchdown during landing (Figure 15). In Figure 15a, takeoff and full stop landing for a flight with LFE=3.27 is shown. In this flight, the performed touchdown was smooth with $LFE_{P}=0.25$; nevertheless, take-off weight of the aircraft was about 2000 kg higher than assumed for simulated landings during FSFT. This caused significant growth of the GAG cycle contribution to the fatigue of landing gear attachment node. The obtained $LFE_{GAG}$ for this flight was 2.84.

**Figure 16:**
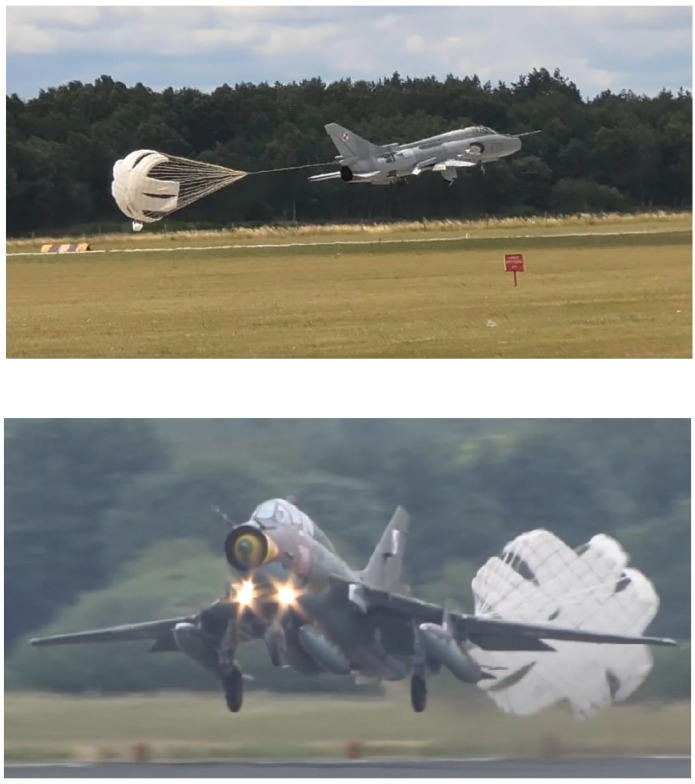
Examples of landings with prematurely opened drogue parachute [29,30,31].

The second example is a flight with hard touchdown during landing with very high LFE=8.37. Take-off weight in this case was comparable, as assumed for FSFT, which resulted in $LFE_{GAG}=1.30$. However, very high load cycle was exerted on the structure during touchdown with the amplitude about 10,000 kg of equivalent carried weight. Thus, $LFE_{P}$ obtained for the flight was 6.95. Such events can be due to a sudden rapid gust of wind or due to the premature release of the drogue parachute (Figure 16), which is sometimes trained as simulation of landing on an airfield with a very short runway.

**Figure 17:**
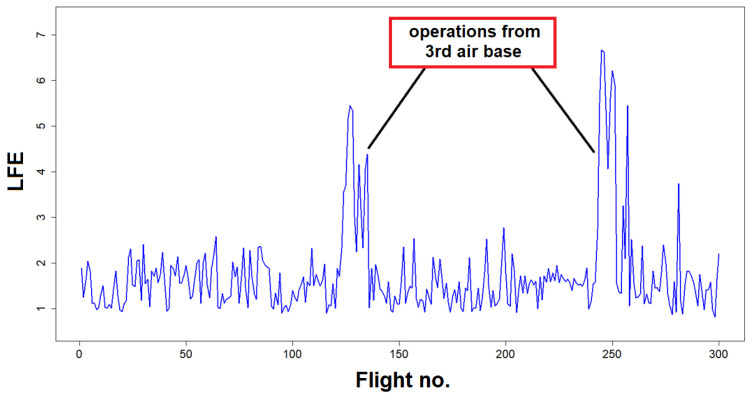
Landing Fatigue Equivalent for subsequent flights of an aircraft in a given period.

Furthermore, interesting cases of increased *LFE* were observed for aircraft operations performed from a particular air base. In Table 5, the total mean *LFE* with contributions of selected type of cycles is presented. Significant increase of fatigue of landing gear attachment node was obtained for aircraft operations from one of the airfields (Figure 17).

**Figure 18:**
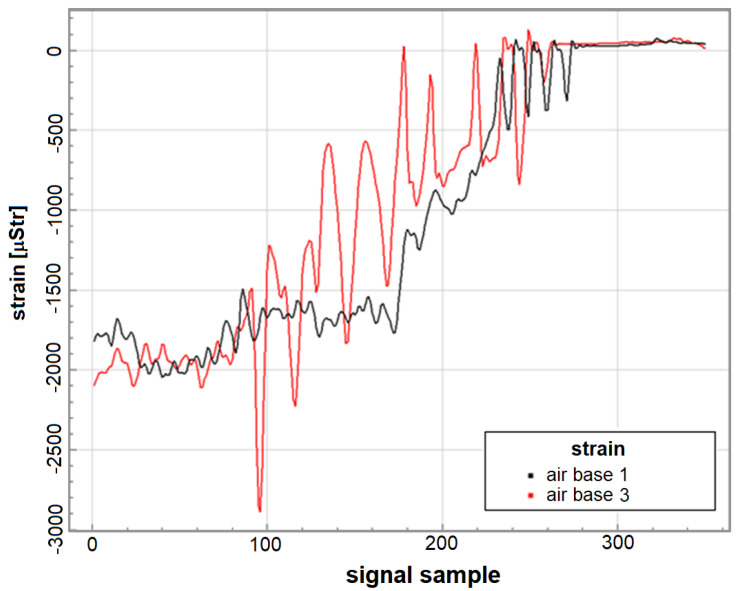
Records of strain during take-off for selected flights from the home base and air base No. 3.

Total mean *LFE* obtained for landings on that airfield was 4.30 compared to fatigue equivalent 1.17 obtained for operations from the home air base. Significant increase of contributions to *LFE* due to different type of cycles was observed for Base 3, in particular *LFE* of cycles due to taxing before take-off, was more than 18 times higher than in regular conditions. In fact, abnormal variation of strain during aircraft take-off was observed as shown in Figure 18, which can be caused by particularly bad condition of runway.

## 4. Conclusions

In this paper, application of operational load monitoring system of an aircraft to fatigue equivalent estimation of landing gear attachment node was presented. In particular, system description algorithms for signal processing and fatigue estimation were provided. The notion of Landing Fatigue Equivalent (*LFE*) was introduced as a measure representing the relative fatigue of landing gear node due to loads exerted during a given landing with respect to mean fatigue of simulated landings during Full-Scale Fatigue Test of the structure. It was demonstrated that the *LFE* obtained for full stop landings is significantly higher than *LFE* for Touch-And-Go landings. The contribution of different types of load cycles to *LFE* was also investigated. Predominant contribution to *LFE*, i.e., over 80%, is due the Ground–Air–Ground (GAG) cycle; furthermore, loads exerted on landing gear attachment node during aircraft touchdown have considerable effect on fatigue wear of the structure. Particular examples of flights with high values of *LFE* were presented and discussed.

## Figures and Tables

**Table 1:** Configurations of aircraft for Full-Scale Fatigue Test.

Stage	Loads	Wing Sweep Angle
I	Landing	30°
II	Flight	45°
III	Flap	30°
IV	Flight with high overloads	45°

**Table 2:** Number of different landings type represented during Full-Scale Fatigue Test.

Landing Type	Landing Count in Load Spectrum
Type 1	1926
Type 2	5603
Type 3	1849
Type 4	589
Type 5	157
Type 6	36
Type 7	9
Type 8	1

**Table 3:** Warning levels for maximum strain amplitude [μStr] during aircraft touchdown.

	Min. Touchdown Amplitude	Max. Touchdown Amplitude
standard landing	0	1100
first warning level	>1100	1500
second warning level	>1500	-

**Table 4:** Contribution of different types of cycles to Landing Fatigue Equivalent (*LFE*).

Cycles Type	GAG	P	D	KL	KS	Other Cycles
median *LFE*	1.040	0.083	0.004	0.030	0.003	0.016

**Table 5:** Mean Landing Fatigue Equivalent values corresponding to different types of cycles with respect to operations performed from different air bases.

Localization	GAG	P	KL	KS	Total
air base No. 1	0.95	0.17	0.04	0.07	1.17
air base No. 2	0.99	0.20	0.03	0.06	1.24
air base No. 3	2.41	0.86	0.18	1.21	4.30

## Data Availability

Data used in this study are available on-demand from the corresponding author.

## Abbreviations

The following abbreviations are used in this manuscript:

FDR	Flight Data Recorder
FSFT	Full Scale Fatigue Test
GAG	Ground–Air–Ground (cycle)
IAT	Individual Aircraft Tracking
*LFE*	Landing Fatigue Equivalent
MLG	Main Landing Gear
OLM	Operational Load Monitoring
T&G	Touch-And-Go (landing)
